# Shedding light on shade-avoidance: *SlPIF8a* plays a pivotal role in the tomato shade response

**DOI:** 10.1093/plcell/koaf091

**Published:** 2025-04-18

**Authors:** Róisín Fattorini

**Affiliations:** Assistant Features Editor, The Plant Cell, American Society of Plant Biologists; Institute of Molecular Plant Sciences, University of Edinburgh, Max Born Crescent, Edinburgh EH9 3BF, UK

Sun-loving plants exhibit remarkable phenotypic plasticity through their shade-avoidance response. This response triggers morphological and physiological changes, such as stem and petiole elongation, that enable the plant to escape suboptimal light-limiting conditions and maximize exposure to the sunlight required for photosynthesis. In the shade, the proportion of far-red light increases and the ratio of red to far-red light (R/FR) decreases. Plant photoreceptors, primarily phytochrome B (phyB), sense shading from neighboring vegetation as a reduction in R/FR. In its active form, phyB inactivates and degrades the transcription factors known as PHYTOCHROME-INTERACTING FACTORS (PIFs) (Qiu et al. 2017). As the R/FR ratio falls below one, phyB is converted into its inactive form, releasing the PIFs from phyB-mediated repression. PIFs regulate multiple processes related to plant growth, including auxin synthesis (Hornitschek et al. 2012; Li et al. 2012; Casal 2013). While the role of PIFs has been well studied in Arabidopsis (*A. thaliana*), little is known about PIF functioning in crop plants despite the potential of PIFs, as growth regulators, to enhance agronomic traits.

In new work in *The Plant Cell*, **Srinivas Kunta and colleagues (Kunta et al. 2025)** found that *SlPIF8a* is a key regulator of the stem elongation response to low R/FR light in tomato (*Solanum lycopersicum*) seedlings. *SlPIF* candidate genes were selected based on high sequence similarity with known regulators in Arabidopsis, *AtPIF4* and *AtPIF7* (Burko et al. 2022). CRISPR-Cas9-generated *slpif* tomato mutants were grown under 1 of 2 light (white or low R/FR) and temperature (21 °C or 30 °C) conditions (Figure). Impaired stem elongation occurred under low R/FR conditions only in plants lacking the wild-type *SlPIF8a* gene (Figure). SlPIF8a functioning was corroborated through additional experiments, including the expression of *SlPIF8a* under its native promoter and a constitutive promoter in *slpif8a* or *slpif* quadruple (*slpif47a7b8a*) mutants, respectively, which led to a partial to nearly full rescue of stem elongation in the *slpif* mutants in low R/FR conditions. Comparisons between the *slpif8a* single mutant and higher order *slpif* double, triple, and quadruple mutants demonstrated that *SlPIF4*, *SlPIF7a*, and *SlPIF7b* also have minor roles in tomato stem elongation during the low R/FR light response.

**Figure.**
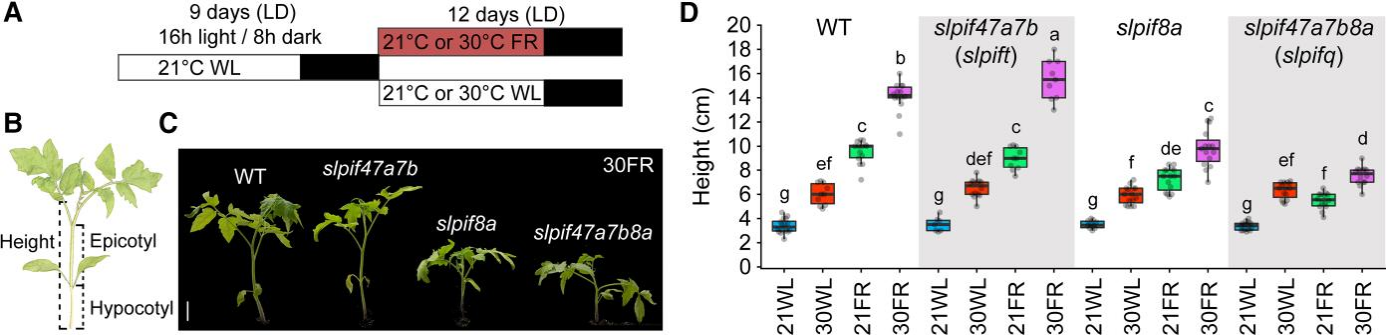
SlPIF8a regulates the elongation response of tomato seedlings to low R/FR at 21 °C and 30 °C. **A)** A diagram of the experimental design for seedling growth: long day conditions (LD), white light (WL), far-red light (FR). **B)** A tomato seedling cartoon defining the areas used for measurements. **C)** Photographs of 21 day old WT and *slpif* mutant tomato seedlings grown as illustrated in A in the 30 °C FR condition. Scale bar = 3 cm. **D)** Height, as illustrated in (B), of WT and *slpif* mutant plants grown in the four conditions illustrated in (A). Different letters indicate statistically significant differences (as determined by 3-way ANOVA and Tukey's HSD, *P* < 0.05) among samples. Adapted from Kunta et al. (2025), Figure 1.

RNA-seq data provided further insight into the dominant role of *SlPIF8a.* A comparison between *SlPIF* expression patterns in the aerial organs of tomato seedlings grown in white light revealed that *SlPIF8a* had the highest expression levels overall, particularly in the cotyledons and first leaf. The low R/FR response is thought to be initiated in these organs with the production of auxin, which is likely transported to the hypocotyl and epicotyl, where it promotes growth. High *SlPIF8a* transcript levels may lead to high protein expression, with SlPIF8a released from phyB-mediated degradation/inactivation upon exposure of the plant to low R/FR conditions. Therefore, the key role of *SlPIF8a* in the stem elongation response to low R/FR light may be, in part, due to its high expression in these organs.

The downstream mechanisms of the tomato low R/FR response were investigated through a series of elegant RNA-seq comparative analyses. A comparison was made between the genes differentially expressed in low R/FR and white light conditions among the wildtype, *slpif8a* single mutant, and *slpif* quadruple mutant (*slpif47a7b8a*) plants. This enabled the identification of genes regulated by both SlPIFs and low R/FR light, which included cell wall modifying genes and genes associated with gibberellin, auxin, and flavonoid biosynthesis. Interestingly, organ-specific expression data revealed that the hypocotyl and epicotyl elongation response mechanisms may be distinct. The response to low R/FR light was associated with the upregulation of cell cycle and cell division-associated genes exclusively in the epicotyl, whereas genes associated with cell elongation were enriched in the hypocotyl. An important next step is to characterize the developmental mechanisms underlying the epicotyl and hypocotyl elongation responses. Subsequently, determining the organ-specific roles of *SlPIFs* in regulating these elongation processes would be an exciting future research avenue.

In conclusion, Kunta et al. provide key insights into the genetic regulation of the stem elongation response to low R/FR light in tomato. The transcription factor SlPIF8a was identified as a key regulator of this process. Future studies could provide an evolutionary perspective by investigating whether *SlPIF8a* gene function is conserved within other Solanum species—a genus containing several important crops.

## Recent related articles in *the plant cell*

- Han et al. (2023) show that SALT OVERLY SENSITIVE2 (SOS2), a protein kinase essential for salt tolerance, positively regulates the shade avoidance response in Arabidopsis, suggesting a coordinated response of plants to salt stress and shade.
- Zhou et al. (2024) show that maize plants readjust their leaf azimuthal orientations in response to high planting density, maximizing canopy light interception.
- Kim et al. (2024) show that differences in the role of promoters, and the molecular activities of proteins, have contributed to functional diversification between *PHYTOCHROME INTERACTING FACTOR 1* (*PIF1*) and *PIF4* in Arabidopsis. The importance of each contributing factor varied depending on the light conditions.

## Data Availability

No new data were generated or analysed in support of this research.

## Dive Curated Terms

The following phenotypic, genotypic, and functional terms are of significance to the work described in this paper:

PIF4 Gramene: AT2G43010
PIF4 Araport: AT2G43010

SlPIF8a Gramene: Solyc01g090790
SlPIF8a Araport: Solyc01g090790
